# Co-Occurrence of Multiple Sclerosis and Amyotrophic Lateral Sclerosis in an FUS-Mutated Patient: A Case Report

**DOI:** 10.3390/brainsci12050531

**Published:** 2022-04-21

**Authors:** Luigi Fiondella, Francesco Cavallieri, Elena Canali, Maria Paola Cabboi, Alessandro Marti, Francesca Sireci, Alena Fiocchi, Gloria Montanari, Sara Montepietra, Franco Valzania

**Affiliations:** 1 Neurology Unit, Neuromotor and Rehabilitation Department, AUSL-IRCCS of Reggio Emilia, 42123 Reggio Emilia, Italy; cava_87@hotmail.it (F.C.); elena.canali@ausl.re.it (E.C.); mariapaola.cabboi@ausl.re.it (M.P.C.); alessandro.marti@ausl.re.it (A.M.); francesca.sireci@ausl.re.it (F.S.); sara.montepietra@ausl.re.it (S.M.); franco.valzania@ausl.re.it (F.V.); 2 Department of Biomedical, Metabolic and Neural Sciences, University of Modena and Reggio Emilia, 41125 Modena, Italy; 3 Clinical and Experimental Medicine PhD Program, University of Modena and Reggio Emilia, 41125 Modena, Italy; 4 Physical Medicine and Rehabilitation Unit, AUSL-IRCCS of Reggio Emilia, 42123 Reggio Emilia, Italy; alena.fiocchi@ausl.re.it; 5 Pneumology Unit, AUSL-IRCCS of Reggio Emilia, 42123 Reggio Emilia, Italy; gloria.montanari@ausl.re.it

**Keywords:** amyotrophic lateral sclerosis, multiple sclerosis, clinical neurophysiology, FUS, oligodendroglial dysfunction

## Abstract

A concomitant presentation of relapsing remitting multiple sclerosis (RRMS) and amyotrophic lateral sclerosis (ALS) is quite rare. However, a review of the literature showed an increased co-occurrence of both diseases, including in genetically determined cases. We report the case of a 49-year-old woman with a history of RRMS who developed a progressive subacute loss of strength in her left arm. The patient’s father died from ALS, and her paternal uncle had Parkinson’s disease. Brain and cervical MRIs were performed, and new demyelinating lesions were excluded. Electromyography (EMG) of the upper limbs showed fibrillations and fasciculations in distal muscles of both arms. In the following months, the patient presented a progressive loss of strength in the proximal and distal muscles of the right arm and hyperreflexia in the lower limbs. EMG and central motor conduction were consistent with ALS. A genetic test was carried out, revealing a mutation in the FUS gene (exon 15; c. 1562 G>A). To our knowledge, the co-occurrence of MS and ALS in patients with FUS mutation is extremely rare. We hypothesize a common pathway for both diseases based on the possibility of a shared oligodendroglial dysfunction due to FUS mutation.

## 1. Introduction

Multiple sclerosis is a primarily inflammatory disorder of the central nervous system (CNS) that causes damage to myelin and axons [1]. In the past few years, the identification of genetic variants affecting the development of the disease has grown almost exponentially, confirming the presence of genetic susceptibility [2]. ALS is a progressive neurodegenerative disorder involving primarily motor neurons in the cerebral cortex, brainstem, and spinal cord. Heterogenous clinical findings early in the disease’s course and the lack of any biological markers make definitive diagnosis difficult [3]. Familial ALS represents ~10% of the total number of ALS cases, and currently more than 30 genes have been linked to familial ALS [4]. The unusual combination of ALS and MS and an increased co-occurrence of ALS and MS has been reported [5,6,7,8,9], also in patients with hexanucleotide repeat expansions of C9ORF72 [10]. These cases suggest a possible common link between neurodegeneration, inflammation, and/or genetic susceptibility. We present the case of a 49-year-old woman with a long history of RRMS who developed concomitant ALS. Due to her young age and familial history of neurodegenerative diseases, a genetic test was performed and an FUS mutation was described.

## 2. Case Presentation

In December 2017, a 49-year-old woman was referred to our Neurology Clinic due to progressive subacute weakness in her left arm, which had started three weeks earlier. The patient had a history of RRMS that started at the age of 25 when she had right optic neuritis preceded a few months earlier by transient urinary hesitancy. The clinical course, the presence of oligoclonal bands in the cerebrospinal fluid, and the brain and cervical MRI (multiple demyelinating lesions in the periventricular and hemispheric deep white matter and cervical spinal cord) all contributed to our diagnosis. She was initially treated with beta interferon. The first relapse occurred in 1994, with dysarthria and sensory loss in her left limbs (Expanded Disability Status Scale, EDSS score: 2.5), while the second relapse occurred in 2000, with sensory loss in her right limbs (EDSS score: 2.5). In 2004, a third relapse led to mild hyposthenia and ataxia in the right limbs and mild urinary hesitancy (EDSS: 3), and azathioprine was introduced. When another relapse took place in 2014, the neurological examination showed mild lower limb hyposthenia with spastic gait, moderate truncal ataxia, moderate urinary urgency, and a mild decrease in mentation (EDSS 4.5). We switched the treatment to fingolimod, but the therapy was suspended early due to leucopenia. Azathioprine was reintroduced. The patient experienced incomplete remission, and the disease course stabilized. During follow-up visits (2016), we observed mild gait ataxia, a positive Romberg test, and mild urinary urgency with an EDSS score of 3.

**Figure 1:**
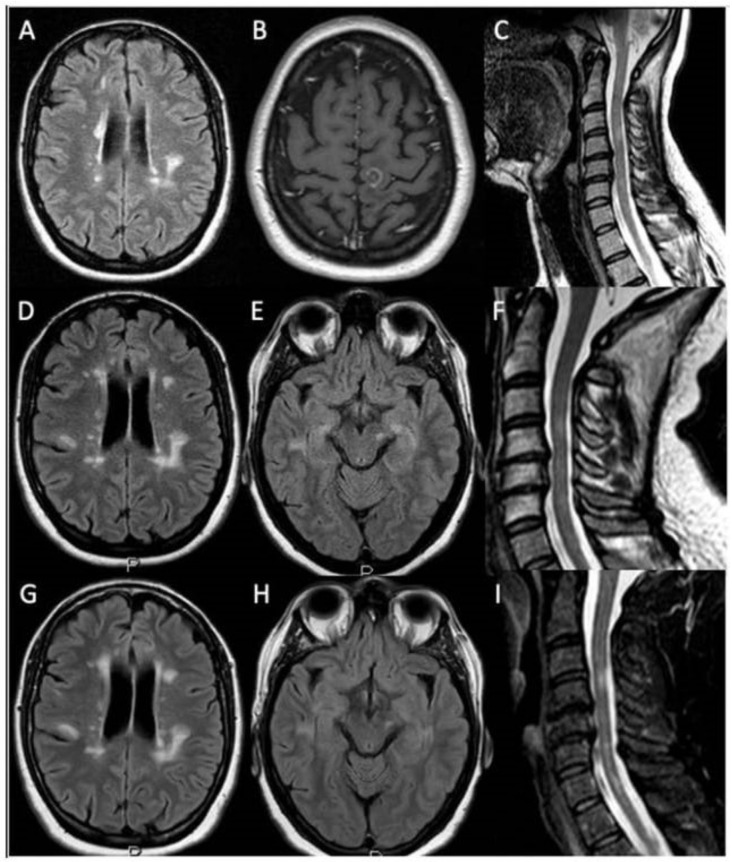
Patient’s brain and spinal cord MRIs. 1.5 tesla brain and spinal cord MRI obtained before and after IV gadolinium injection. November 2006: axial fluid-attenuated inversion recovery (FLAIR) and t2 weighted sequences showed the presence of multiple demyelinating lesions located in the periventricular and hemispheric deep white matter (**A**) and cervical spinal cord (**C**), including one contrast enhancing lesion in the left fronto-parietal juxtacortical white matter (**B**). November 2011: FLAIR and t2 weighted sequences showed the presence of multiple demyelinating lesions within the periventricular and hemispheric deep white matter (**D**), left mesencephalic (**E**) and cervical spinal cord regions (**F**). February 2018: brain MRI FLAIR sequences showed the presence of multiple demyelinating lesions located in the periventricular and hemispheric deep white matter (**G**) and left mesencephalic region (**H**) (superimposable on previous MRI studies) without new contrast enhancing lesions. Cervical spinal cord t2 weighted sequences displayed the presence of multiple demyelinating lesions (unchanged with respect to the previous MRI study) together with cervical spondylosis with posterior osteophytosis without spinal cord and nerve roots compression (**I**).

The patient’s father died from ALS and her paternal uncle had Parkinson disease (genetic test was not performed in both patients). The medical history was otherwise unremarkable, and at the time of presentation she was not taking medication other than azathioprine. In December 2017, neurological examination revealed hypotrophy of the left deltoid, the left periscapular, and the left bicep. Weakness of the following muscle was found (right/left; Medical Research Council [MRC] grades): deltoids (5/3), supraspinatus (5/4), infraspinatus (5/4), biceps (5/4−), finger extensor (5/3). Interosseous, finger flexors, wrist extensor/flexor, brachioradialis, triceps were 5/5. Tendon reflexes were (right/left): triceps (2+/0), biceps (2+/1+), brachioradialis (2+/1+), Achilles’ tendon and patellar reflex (2+/2+). We also observed mild sensory ataxia, with a positive Romberg test. A brain MRI was performed, and new demyelinating lesions were not shown (Figure 1G,H). Considering the prominent involvement of the left upper limb, a cervical MRI was performed and detailed spondylosis with posterior osteophytosis without spinal cord and nerve root compression (Figure 1I).

Nerve conduction studies (NCS) of the upper limbs showed normal compound motor action potential (cMAP) in the right arm but reduced cMAP of all the tested motor nerves on the left side (ulnar, median, musculocutaneous, and circumflex). Sensory action potentials (SAP) were spared. The F waves were increased in amplitude and of low persistence in the left arm. Needle EMG showed fasciculations and fibrillations in first interosseous muscles and biceps bilaterally.

In the following months, the patient complained of a progressive loss of strength in the proximal and distal muscles of the right arm. At that time, neurological examination revealed bilateral deltoids, supraspinatus, infraspinatus, and left biceps atrophy. We observed fasciculations in the atrophic muscles and in the lower limbs. Weakness of the following muscle was found (right/left; Medical Research Council [MRC] grades): deltoids (3/2), supraspinatus (4/3), infraspinatus (4/3), biceps (5/4−), finger extensor (5/3). Interosseous, finger flexors, wrist extensor/flexor, brachioradialis, triceps were 5/3. Tendon reflexes were symmetrically hyperactive without clonus in upper and lower limbs. Bilateral Babinski sign and Hoffmann sign were found on the left arm.

**Figure 2:**
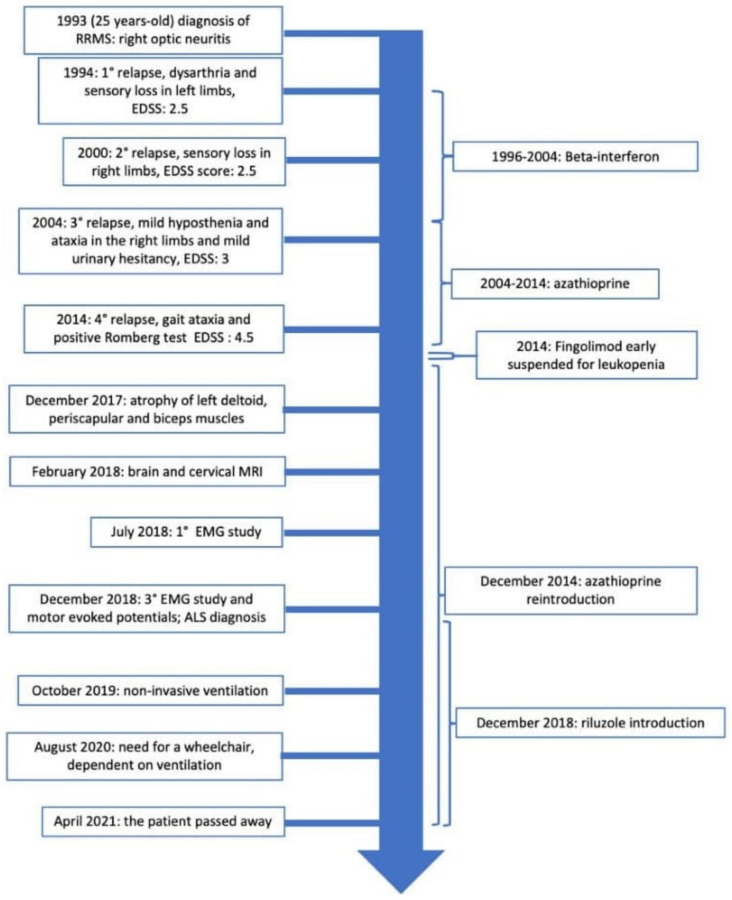
Timeline of the patients history.

EMG was repeated, indicating the presence of fasciculation and fibrillation potentials together with neurogenic signs in different districts (right deltoid, right trapezius, first interosseous and biceps bilaterally; right gastrocnemius, tibialis, and vastus medialis bilaterally). In addition, NCS showed reduced cMAP in all the motor nerves in the left arm without any sensory involvement. Motor evoked potentials showed an increased central conduction time in the four limbs. Blood count, complete metabolic panel, HbA1C, serum protein electrophoresis/immunofixation electrophoresis, C-reactive protein, anti-MAG, anti-GAD, onconeural antibodies, antinuclear antibodies, and a total body CT scan were all normal. The patient underwent a lumbar puncture and the isoelectrofocusing observed the presence of oligoclonal bands. A diagnosis of amyotrophic lateral sclerosis was made (3). In the light of the familial history of ALS, a genetic test was performed, based on a customized next-generation sequencing panel (NGS, Custom Panel Enrichment, Nextera Flex Enrichment-Illumina) including all the coding exons and the flanking intron-exon boundaries of 24 genes (ALS2, ANG, CHMP2B, DCTN1, FUS, GRN, HNRNPA1, HNRNPA2B1, MAPT, MATR3, NEK1, OPTN, PFN1, SETX, SOD1, SPAST, SPG11, SQSTM1, TARDBP, TBK1, TUBA4A, UBQLN2, VAPB, VCP) together with fluorescent and repeat-primed PCR (RP-PCR) analysis of pathological expansion of the GGGGCC hexanucleotide in C9ORF72 gene, revealing a heterozygous mutation in the FUS exon 15 (c. 1562 G>A) [11,12]. A multidisciplinary team (two neurologists, a pneumologist, a physiatrist, and a psychologist) worked to provide integrated care and monitored the patient for the next two years. In October 2019, noninvasive ventilation was started; at that time the patient was still able to walk without ambulatory assistive devices, but her upper limbs were plegic. By 2020, she needed to use a wheelchair, and she became dependent on ventilation for most of the day. Palliative care and dignity therapy began, and the patient died in April 2021. Figure 2 shows the timeline of the events.

## 3. Discussion

This patient presented with a subacute loss of strength in her left arm, associated with homolateral proximal atrophy, triceps areflexia, and biceps/brachioradialis hyporeflexia. Asymmetric weakness most likely reflects disease of the central or peripheral nervous system. A relapse of RRMS must be ruled out despite the disease’s extensive history. Among the central causes of muscular atrophy, amyotrophy combined with parietal lesion (Silverstein’s Syndrome) must be considered [13]. However, the pattern of focal muscle involvement, the non-pyramidal distribution, and the lack of corticospinal tract signs argue against a CNS disease. Focal muscular atrophy, weakness, and areflexia can arise from several conditions that affect the lower motor neurons. The most common include focal spinal lesions, traumatic root lesions, plexus injuries, brachial plexopathies, mononeuropathies due to different causes, multifocal motor neuropathy, monomelic amyotrophy, early motor neuron disease, and variants of spinal muscular atrophy (SMA).

The pattern of weakness in this patient involved different muscles in the distribution of several myotomes (from C5 to C8) and different motor nerves. Focal spinal lesions, cervical root lesions, or brachial plexopathy could have a similar presentation, but the absence of pain and sensory symptoms made these unlikely. Multifocal motor neuropathy with conduction block is characterized by an asymmetric subacute onset, typically with involvement of the distal muscles of the upper limbs [14]. Brachial monomelic amyotrophy (Hirayama Disease) primarily affects young males and is distinguished by preferential weakness and atrophy affecting intrinsic muscles of the hand and forearm muscles, and is usually confined to a single limb [15]. At the initial stage, the absence of clinical upper motor neuron signs overshadows the diagnosis of ALS. Based on the clinical data, we performed a brain and cervical MRI that excluded new demyelinating lesions. The results of the upper limbs’ EMG ruled out several differential diagnoses of lower motoneuron disease spectrum disorders. ALS was suspected since neurogenic signs associated with focal muscle involvement were also found in the contralateral arm. This suspicion was confirmed during the subsequent follow-up.

In patients with MS, lower motor neuron dysfunction associated with spinal cord plaques has been observed. However, we considered that the rapid progression of the motor signs, as well as the abrupt change in the previous disease course, are more characteristic of ALS than MS, even in its progressive forms.

Given the family history, a genetic test was performed and an FUS mutation (c. 1562 G>A) was found. Over 50 mutations on the FUS gene have been discovered in familial and sporadic ALS patients. The majority of these mutations were heterozygous and passed down through autosomal dominant inheritance [11,16,17]. Mutations in the FUS gene cause aggressive, sometimes juvenile-onset disease (17). Rare co-occurrence of RRMS has been reported in ALS-FUS-mutated patients [18,19], but FUS mutations are not thought to be causative of multiple sclerosis. We note that only two cases of concomitant RRMS and ALS in FUS-mutated patients have been reported in the literature to date. The first was a woman with a long history of RRMS who developed bulbar-onset ALS at the age of 62. ALS onset was characterized by a 14-month history of bulbar symptoms (i.e., dysphagia, dysarthria, and facial weakness) associated with the right hand, and progressive weakness and atrophy in the legs. This case carried a p.Gly174del. in exon 5 of the FUS gene [18].

The second patient was a 45-year-old woman affected by MS with predominantly cerebellar and sensory involvement, which was validated by neuroradiological and CSF investigations (intrathecal oligoclonal IgG synthesis). ALS started insidiously through progressive upper limb weakness (prevalent on the left side) together with diffuse fasciculations and intrinsic hand-muscle atrophy. Afterwards, dysphagia, dysphonia, and progressive weakness of the four limbs developed. In this case, the patient carried a P525L substitution in exon 15 of the FUS gene [19]. The concurrence of MS/ALS has been reported in more than 30 cases (Table 1).

In almost all cases, patients demonstrated sequential disease occurrence, with MS preceding ALS. The earlier age of onset of MS relative to ALS could explain this evidence. Furthermore, MS therapies have resulted in longer life expectancies, allowing the subsequent development of other diseases, including ALS [26]. Considering the incidence rates, the incidental occurrence of both diseases should be an exceptional event. However, we may hypothesize that between both illnesses there exists a possible common pathway. For instance, oligodendroglial dysfunction and cell death are features of different human CNS diseases, such as demyelinating diseases, traumatic brain and spinal cord injuries, or neurodegenerative diseases. Of note, oligodendrocyte (OL) damage and the impaired maturation of OL precursor cells in ALS share similarities with progressive forms of MS, where OL pathology and myelin deterioration can lead to neuronal death [27]. Particularly, the authors proposed several causes for OL pathology: ALS-causing mutant genes, reactive glial cells, oxidative damage, glutamate-mediated excitotoxicity, and alterations in gene expression. As a result, different insults contribute together to OL dysfunction that leads to secondary neurodegeneration. Several studies have confirmed that OL pathology can initiate axonal damage. For instance, mouse models with genetically induced OL death display increasing motor impairment and severe axonal degeneration [28,29,30]. In ALS-FUS-mutated patients, myelin damage has been associated with cytoplasmic inclusions of mutant FUS aggregates in mature OLs, primarily contributing to functional deficits [31,32]. Scekic-Zahirovic et al. characterized an FUS heterozygous knock-in mouse model, in which the FUS gene is truncated, and demonstrated a downregulation of genetic encoding for proteins related to myelination (myocilin, Ncmap, Pmp2, Pmp22, and Cldn19). Consistent with these data, ventral root axon calibers were modestly reduced and only a few axons showed normal myelination. Finally, muscles revealed an increased latency of CMAP potentials, which contributed to motor deficits in mice [32]. Accordingly, a recent work described the crucial role of FUS in controlling myelination by exploiting conditional OL-specific FUS knockout mice [33]. 

This study discovered that FUS has a role in regulating myelin deposition, which is linked to enhanced activation of the Akt serine/threonine kinase pathway and expression of the rate-limiting enzyme involved in cholesterol biosynthesis (3-hydroxy-3-methylglutaryl-coenzyme A reductase, HMGCR). In vivo, Akt overexpression in OLs increases myelin production rather than cell survival or proliferation. The authors propose that FUS loss of function raises HMGCR, resulting in higher cholesterol, which leads to expanded myelin synthesis and activation of Akt through lipid platform assembly. This evidence confirmed the hypothesis of FUS effect on myelin deposition control. On this premise, we can speculate that in our case, both MS and ALS share a common oligodendroglial dysfunction due to FUS mutation, which contributes to the clinical phenotype. Further studies are needed to confirm this hypothesis.

## 4. Conclusions

We presented the case of a patient affected by RRMS who developed concomitant ALS with FUS gene mutation. To our knowledge, the co-occurrence of both diseases in patients with FUS mutation is extremely rare [18,19]. Considering the epidemiology of MS, the overlap with ALS could be coincidental, although there is evidence of a possible common pathway due to oligodendroglial dysfunction. Finally, our case reinforces the relevance of anamnestic information (the familial history in this specific case), which is always relevant in the diagnostic process. Moreover, a long history of illness does not exclude the possibility of other concomitant rare diseases, even when at first glance it may seem a typical clinical presentation or, as in our case, a relapse. We suggest vigilance to avoid delay in diagnosis.

## Figures and Tables

**Table 1:** Demographics and clinical features of previously described MS-ALS patients.

Reference	Multiple Sclerosis				ALS	
	Gender	AO	SO	Form	AO	Site of Onset
Confavreux [20]	F	25	Hemiparesis	RRMS	35	Limb
Li [21]	M	37	VI nerve palsy	SPMS	39	Bulbar
Hader [9]	M	21	UNK	RRMS	56	Bulbar
Borisow [8]	M	UNK	UNK	UNK	55	Limb
Machner [22]	F	55	Gait disorder	UNK	56	Limb
Dynes [6]	F	61	UNK	UNK	61	Limb
Trojsi [7]	F	33	UNK	PPMS	34	Limb
Ismail [10]	F	22	UNK	SPSM	62	Bulbar
Ismail	F	49	UNK	RRMS	52	Bulbar
Ismail	F	43	UNK	RRMS	52	Limb
Ismail	M	46	UNK	PPMS	67	Bulbar
Ismail	M	39	UNK	PPMS	40	Bulbar
Ismail	F	40	Pain-weakness upper limbs	SPMS	41	Limb
Ismail	F	56	Left upper limb and lower limbs	PPMS	56	Limb
Dattola [23]	F	UNK	UNK	UNK	47	UNK
Dattola	F	35	Diplopia	RRMS	38	Bulbar
Dattola	F	UNK	UNK	RRMS	52	Bulbar
Dattola	F	25	UNK	RRMS	49	UNK
Guennoc [24]	F	41	Optic neuritis	SPMS	52	Bulbar
Guennoc	F	39	Sensory	SPMS	51	Limb
Guennoc	M	34	Sensory	SPMS	50	Limb
Guennoc	F	41	Weakness lower limbs	PPMS	66	Limb
Guennoc	F	27	Optic neuritis	RRMS	59	Limb
Guennoc	F	54	Spastic gait	PPMS	55	Limb
Allen [25]	M	27	Sensory	RRMS	51	Limb
Hewitt [18]	F	UNK	UNK	RRMS	62	Bulbar
Sproviero [19]	F	UNK	Sensory	RRMS	45	Limb
Pocock [26]	F	UNK	Right leg weakness	RRMS	70	Limb
Pocock	F	49	UNK	RRMS	72	Bulbar
Pocock	F	44	UNK	SPMS	51	Bulbar
Pocock	F	49	Left leg and arm	PPMS	49	Limb
Pocock	M	UNK	UNK	SPMS	63	Bulbar

AO: age of onset (years); SO: symptoms of onset; UNK: unknown; SPMS: secondary progressive MS; PPMS: primary progressive multiple sclerosis; RRMS: remittent relapsing MS; ALS: amyotrophic lateral sclerosis.

## Data Availability

The authors confirm that the data supporting the findings of this study are available within the article.
